# Supplementary material for: Multi‐dataset identification of innovative feature genes and molecular mechanisms in keratoconus
Source: J Cell Mol Med. 2024 Sep 19;28(18):e70079. doi: 10.1111/jcmm.70079 (PMC11412914; doi:10.1111/jcmm.70079)
Supplement: Supplementary file 2 — Material S2. [file JCMM-28-e70079-s002.zip › 7.GSEA analysis/1.ARL11/KEGG_FC_EPSILON_RI_SIGNALING_PATHWAY.html]

Details for gene set KEGG\_FC\_EPSILON\_RI\_SIGNALING\_PATHWAY[GSEA]

|  || Dataset | ARL11.group.cls#h\_versus\_l |
| Phenotype | group.cls#h\_versus\_l |
| Upregulated in class | l |
| GeneSet | KEGG\_FC\_EPSILON\_RI\_SIGNALING\_PATHWAY |
| Enrichment Score (ES) | -0.3004231 |
| Normalized Enrichment Score (NES) | -1.0691336 |
| Nominal p-value | 0.3824131 |
| FDR q-value | 1.0 |
| FWER p-Value | 0.998 |
Table: GSEA Results Summary

  

Fig 1: Enrichment plot: KEGG\_FC\_EPSILON\_RI\_SIGNALING\_PATHWAY      
 Profile of the Running ES Score & Positions of GeneSet Members on the Rank Ordered List

  

| SYMBOL | TITLE | RANK IN GENE LIST | RANK METRIC SCORE | RUNNING ES | CORE ENRICHMENT || 1 | PIK3R3 | na | 2180 | 0.284 | -0.0287 | No |
| 2 | PIK3CG | na | 2870 | 0.247 | -0.0188 | No |
| 3 | IL3 | na | 3483 | 0.220 | -0.0099 | No |
| 4 | PLCG1 | na | 4334 | 0.189 | -0.0116 | No |
| 5 | RAC3 | na | 4612 | 0.179 | 0.0019 | No |
| 6 | FCER1A | na | 4733 | 0.174 | 0.0195 | No |
| 7 | PLA2G4E | na | 4751 | 0.174 | 0.0399 | No |
| 8 | INPP5D | na | 5042 | 0.165 | 0.0515 | No |
| 9 | PIK3R1 | na | 6079 | 0.135 | 0.0379 | No |
| 10 | PLCG2 | na | 6096 | 0.135 | 0.0537 | No |
| 11 | SOS1 | na | 6194 | 0.132 | 0.0668 | No |
| 12 | PLA2G3 | na | 6310 | 0.129 | 0.0790 | No |
| 13 | FCER1G | na | 7143 | 0.109 | 0.0681 | No |
| 14 | VAV2 | na | 8664 | 0.080 | 0.0339 | No |
| 15 | PIK3R2 | na | 8783 | 0.078 | 0.0400 | No |
| 16 | PLA2G12B | na | 9215 | 0.072 | 0.0362 | No |
| 17 | AKT2 | na | 9348 | 0.070 | 0.0407 | No |
| 18 | SOS2 | na | 9955 | 0.061 | 0.0306 | No |
| 19 | VAV3 | na | 9977 | 0.060 | 0.0373 | No |
| 20 | IL13 | na | 10076 | 0.059 | 0.0416 | No |
| 21 | PIK3R5 | na | 10118 | 0.059 | 0.0475 | No |
| 22 | LCP2 | na | 10211 | 0.058 | 0.0518 | No |
| 23 | PRKCA | na | 10617 | 0.052 | 0.0464 | No |
| 24 | KRAS | na | 10630 | 0.052 | 0.0524 | No |
| 25 | PDPK1 | na | 10673 | 0.052 | 0.0574 | No |
| 26 | MAPK9 | na | 11167 | 0.046 | 0.0487 | No |
| 27 | MAPK11 | na | 11276 | 0.044 | 0.0509 | No |
| 28 | MAPK12 | na | 13078 | 0.026 | 0.0021 | No |
| 29 | PLA2G2C | na | 13424 | 0.024 | -0.0050 | No |
| 30 | PIK3CA | na | 13512 | 0.023 | -0.0048 | No |
| 31 | JMJD7-PLA2G4B | na | 14509 | 0.015 | -0.0317 | No |
| 32 | MAP2K7 | na | 14659 | 0.014 | -0.0344 | No |
| 33 | MAPK10 | na | 15299 | 0.009 | -0.0517 | No |
| 34 | RAC1 | na | 16390 | 0.001 | -0.0830 | No |
| 35 | LAT | na | 16728 | -0.001 | -0.0926 | No |
| 36 | PRKCD | na | 16865 | -0.002 | -0.0962 | No |
| 37 | MAP2K2 | na | 16997 | -0.003 | -0.0996 | No |
| 38 | PLA2G4A | na | 17245 | -0.005 | -0.1062 | No |
| 39 | MAPK14 | na | 17567 | -0.007 | -0.1145 | No |
| 40 | PLA2G6 | na | 17692 | -0.008 | -0.1171 | No |
| 41 | MAP2K4 | na | 17706 | -0.008 | -0.1165 | No |
| 42 | PLA2G12A | na | 17822 | -0.009 | -0.1187 | No |
| 43 | AKT1 | na | 18085 | -0.011 | -0.1250 | No |
| 44 | HRAS | na | 18435 | -0.013 | -0.1335 | No |
| 45 | RAC2 | na | 18868 | -0.016 | -0.1440 | No |
| 46 | SYK | na | 19461 | -0.020 | -0.1586 | No |
| 47 | PLA2G2E | na | 19951 | -0.023 | -0.1699 | No |
| 48 | VAV1 | na | 20088 | -0.024 | -0.1709 | No |
| 49 | MAP2K3 | na | 20612 | -0.028 | -0.1826 | No |
| 50 | MAPK13 | na | 20970 | -0.031 | -0.1892 | No |
| 51 | MAPK3 | na | 22614 | -0.046 | -0.2310 | No |
| 52 | TNF | na | 22702 | -0.047 | -0.2278 | No |
| 53 | GRB2 | na | 23043 | -0.051 | -0.2315 | No |
| 54 | RAF1 | na | 23336 | -0.054 | -0.2335 | No |
| 55 | PLA2G10 | na | 23788 | -0.060 | -0.2393 | No |
| 56 | PIK3CB | na | 23818 | -0.060 | -0.2329 | No |
| 57 | MAPK8 | na | 24525 | -0.070 | -0.2448 | No |
| 58 | MAPK1 | na | 24626 | -0.071 | -0.2391 | No |
| 59 | PIK3CD | na | 25759 | -0.087 | -0.2614 | No |
| 60 | LYN | na | 25913 | -0.089 | -0.2551 | No |
| 61 | FYN | na | 26579 | -0.100 | -0.2622 | No |
| 62 | AKT3 | na | 27547 | -0.120 | -0.2756 | No |
| 63 | NRAS | na | 27790 | -0.126 | -0.2675 | No |
| 64 | PLA2G2D | na | 27985 | -0.130 | -0.2573 | No |
| 65 | MAP2K6 | na | 28948 | -0.155 | -0.2663 | No |
| 66 | CSF2 | na | 30130 | -0.191 | -0.2773 | Yes |
| 67 | GAB2 | na | 30132 | -0.192 | -0.2542 | Yes |
| 68 | PLA2G2F | na | 30623 | -0.208 | -0.2432 | Yes |
| 69 | MAP2K1 | na | 31070 | -0.223 | -0.2292 | Yes |
| 70 | BTK | na | 31389 | -0.235 | -0.2101 | Yes |
| 71 | PLA2G5 | na | 32093 | -0.264 | -0.1985 | Yes |
| 72 | PRKCE | na | 32224 | -0.270 | -0.1696 | Yes |
| 73 | PLA2G2A | na | 32310 | -0.275 | -0.1389 | Yes |
| 74 | IL5 | na | 32563 | -0.292 | -0.1110 | Yes |
| 75 | MS4A2 | na | 32620 | -0.296 | -0.0769 | Yes |
| 76 | PLA2G1B | na | 33526 | -0.362 | -0.0594 | Yes |
| 77 | IL4 | na | 33709 | -0.380 | -0.0188 | Yes |
| 78 | PRKCB | na | 33863 | -0.399 | 0.0250 | Yes |
Table: GSEA details [plain text format]

  

Fig 2: KEGG\_FC\_EPSILON\_RI\_SIGNALING\_PATHWAY      
 Blue-Pink O' Gram in the Space of the Analyzed GeneSet

  

Fig 3: KEGG\_FC\_EPSILON\_RI\_SIGNALING\_PATHWAY: Random ES distribution      
 Gene set null distribution of ES for **KEGG\_FC\_EPSILON\_RI\_SIGNALING\_PATHWAY**
